# Supplementary material for: Rapid turnover of life-cycle-related genes in the brown algae
Source: Genome Biol. 2019 Feb 14;20:35. doi: 10.1186/s13059-019-1630-6 (PMC6374913; doi:10.1186/s13059-019-1630-6)
Supplement: Supplementary file 3 — Protein alignments, corresponding Gblocks html file and CDS sequences for the lineage conserved gametophyte-biased genes. (ZIP 2109 kb) [file 13059_2019_1630_MOESM3_ESM.zip › Con_GA_alignments/Ec-26_000940.aa_ali.fasta-gb.htm]

Ec-26\_000940.aa.fasta.trans.aa\_ali.fasta


## Gblocks 0.91b Results

Processed file: **Ec-26\_000940.aa.fasta.trans.aa\_ali.fasta**  
Number of sequences: **2**  
Alignment assumed to be: **Protein**  
New number of positions: **279** (selected positions are underlined in blue)

```
                         10        20        30        40        50        60
                 =========+=========+=========+=========+=========+=========+
Ec-26_000940     MQAPAVRHPSPRPPQQQQQQHLNGPINNPHGGLLLRPHPAPGGPSRPALLAPDLQLPVKV
MSTRG.18494.1    -------------------------XTGVHGDLPGHPNPS-MAIARGVHKGPAWPRPFEL
                                              ##########      ###############


                         70        80        90       100       110       120
                 =========+=========+=========+=========+=========+=========+
Ec-26_000940     RPAAPRGVAGVGPATAVNASSWLLKGAAGVPPAMQGSGHKQEGVLRRHRPPSLCGGGTPV
MSTRG.18494.1    RLAAMSRHTGTTPAAGMNASS-LMKIKADQDVRPHGNKYLQARPRGGCPLGAGADGCEVI
                 ##################### ###################              #####


                        130       140       150       160       170       180
                 =========+=========+=========+=========+=========+=========+
Ec-26_000940     ASGPHVFTAGSVGRGARPMLSSNTGRHTQRAYNPIQTRPAEGSRSPNQGALAPERPSCGT
MSTRG.18494.1    YPGPFLPIAGSIARGVGPMRSPEAIRRGQEKLSGTDIRQADLPRSSSYESLASEHSFHSA
                 ############################################################


                        190       200       210       220       230       240
                 =========+=========+=========+=========+=========+=========+
Ec-26_000940     VSQGSVLSNPVGSQQQLRKYAKPPRPPGPPPPHIIEEQRRRREMLAAQAAEEKIASPHHR
MSTRG.18494.1    LSQASGCSIIGAHKRQTRKHVKPPRPPGPPPPHILEEQRKRRELMAQQKMQQHSAYTSTK
                 #######################################################     


                        250       260       270       280       290       300
                 =========+=========+=========+=========+=========+=========+
Ec-26_000940     VAEREPGRAISP-----------A-----------------SP-----------------
MSTRG.18494.1    AGDADAGEGNVPGARGGWDGGHRESPRHGAAKERSRPASLEFPCDTRQQSIVSTGRRDRI
                                                                             


                        310       320       330       340       350       360
                 =========+=========+=========+=========+=========+=========+
Ec-26_000940     --RNTHLVQAGANILSTGSGYGLHMDSNAPLGMGGGGAGATAAATVSTSSASG-----PL
MSTRG.18494.1    GGSTTHPVRAGASILTNGESSDLREYSGRPSGITIQSNEAASPALISQEPTGGRGQNSSR
                     #################################################       


                        370       380       390       400       410       420
                 =========+=========+=========+=========+=========+=========+
Ec-26_000940     SLAPSSCKGSDINP-------------PSTPSCKEWGEFWDEEVGASYYYNSVTGEALWV
MSTRG.18494.1    SLPPVVRTSPDPGPKDLILSGQCANGSSRARSPPRWREYWDEEVEASYYYNSVTREARWV
                 ##############                 #############################


                 
                 =====
Ec-26_000940     RPDGF
MSTRG.18494.1    PPEEL
                 ##
```

```
Parameters used
Minimum Number Of Sequences For A Conserved Position: 2
Minimum Number Of Sequences For A Flanking Position: 2
Maximum Number Of Contiguous Nonconserved Positions: 8
Minimum Length Of A Block: 10
Allowed Gap Positions: None
Use Similarity Matrices: Yes
```

```
Flank positions of the 7 selected block(s)
Flanks: [30  39]  [46  81]  [83  101]  [116  235]  [305  353]  [361  374]  [392  422]  

New number of positions in Ec-26_000940.aa.fasta.trans.aa_ali.fasta-gb:  279  (65% of the original 425 positions)
```
